# Natural spices used for culinary purposes in Nigeria: vendor practices, bacterial contamination, and antimicrobial susceptibility patterns

**DOI:** 10.3389/fmicb.2026.1779848

**Published:** 2026-03-20

**Authors:** Uzochukwu G. Ekeleme, Ijeoma G. Chukwuemeka, Queeneth C. Onuoha, Christopher Chike A. Okereke, Chidinma O. Akanazu, Chiamaka C. Ogujiuba, Chigozie C. Ukachukwu, Ugonma Winnie Dozie, Chinwendu L. Opara, Amarachi B. Nwokoro, Ezejindu Cosmas Nnadozie, Juliana Chinyere Omire, Uchechukwu M. Chukwuocha

**Affiliations:** 1 Department of Public Health, School of Health Technology, Federal University of Technology, Owerri, Imo State, Nigeria; 2 Department of Public Health, Faculty of Allied Health Sciences, David Umahi Federal University of Health Sciences, Uburu, Ebonyi State, Nigeria

**Keywords:** antimicrobial susceptibility, bacterial contamination, natural spices, resistance, vendor practices

## Abstract

**Introduction:**

The increasing demand for natural spices used for culinary purposes in Nigerian local markets has raised concerns about the potential contamination of these products by harmful microorganisms. This study investigated natural spices used for culinary purposes in Nigeria by assessing vendor practices, bacterial con-tamination, and antimicrobial susceptibility patterns.

**Methods:**

A cross-sectional design was employed, with a sample of 267 vendors selected from a population of 800 spice sellers via stratified random sampling. Samples of commonly sold natural spices, including cloves, cinnamon, turmeric, rosemary, basil, and others, were collected for microbiological analysis. Standard microbiological techniques were used to isolate and identify the bacteria present, followed by antimicrobial susceptibility testing via the disc diffusion method.

**Results:**

The results revealed that common storage practices include open air (40%) and covered containers (32%). Handling was mostly done with bare hands (45%), and only 15% used gloves. While 60% of vendors practice hygiene, only 15% receive training on spice handling. The quality control was performed primarily through visual inspection (50%). The viable count of 1.2 × 10^4^ cfu/g, a coliform count of 5.0 × 10^2^ cfu/g indicates relatively low contamination and a bacterial count of 3.4 × 10^3^ cfu/g suggests a fairly high bacterial presence. Cinnamon, on the other hand, presented the lowest levels of bacterial contamination, with a TVC of 0.1 × 10^4^ cfu/g, a coliform count of 1.0 × 10^2^ cfu/g and a bacterial count of 1.5 × 10^3^ cfu/g, further indicating that cinnamon is less prone to microbial growth. Turmeric had the highest microbial loads, with a TVC of 3.4 × 10^4^ cfu/g, a coliform count of 8.0 × 10^2^ cfu/g, and a bacterial count of 4.0 × 10^3^ cfu/g. The presence of various bacteria, including species of *Escherichia coli* and *Staphylococcus aureus*, among others, was revealed. Antimicrobial susceptibility testing revealed that *S. aureus* exhibited significant resistance to penicillin, whereas *E. coli* presented resistance to common antibiotics such as ampicillin and tetracycline.

**Discussion:**

These findings suggest the presence of potential antimicrobial-resistant bacteria in natural spices, indicating the need for improved hygiene practices among spice vendors.

## 1 Introduction

Natural spices such as cloves, cinammon, turmeric, coriander seeds, rosemary, cumin, nutmeg and ginger are in use because of their nutritional, medical effects, thus they appear to be a crucial element in diets across the world (Alegbeleye et al., 2022). These spices are frequently used in the cooking process in Nigeria and they are mostly bought in the open markets thus subjecting them to contamination (Kyrylenko et al., 2023). Although spices add taste and are beneficial to health (Sulieman et al., 2023), they also bring with them dangerous microorganisms that cause foodborne illnesses (Cicero et al., 2022). Generally, due to this concern, this study is to evaluate vendor practices, bacterial contamination and antimicrobial susceptibility patterns of bacteria found in natural spices sold in the Abuja Municipal Area Council (AMAC) of Nigeria.

Microbial contamination of spices is a known phenomenon world over (Wang et al., 2025). Samad et al. (2023) and Ababneh et al. (2023) have reported spices to be contaminated with bacteria and fungi. This has also created great alarm concerning food safety and antimicrobial resistance (AMR) (World Health Organization (WHO), 2022). In Nigeria, microbial loads have been found to be high in industrially packed food seasonings implying that during handling and storage of the food, poor practices obtain (Alegbeleye et al., 2022).

Increased AMR is a significant health care issue (Wang et al., 2025), and the WHO has raised alarms of rising mortality and morbidity levels caused by drug-resistant pathogens (World Health Organization (WHO), 2022; Ahmed et al., 2024). By 2019, there were 929,000 deaths as a result of the AMR, and it is projected that in 2050 there are up to 10 million deaths annually (Naghavi et al., 2023; Salam et al., 2023). The fact that natural spices harbour antimicrobial-resistant bacteria is critical in this discussion bearing in mind that its contamination can facilitate the dissemination of resistant pathogens (Kuforiji et al., 2024).

Although there has been an increasing understanding of the microbial contamination of spices, not much research has been done on the species of the bacteria that is present in the spices used in Nigeria and their antimicrobial resistance. Studies that have been conducted have been more biased to other parts of the world, not knowing that most countries mainly do activities specific to them, including the level of contamination attributed to the spices in Nigeria. Thus, to cover this knowledge gap, this study aims vendor practices, bacterial contamination and antimicrobial susceptibility profile of bacterial associated with spices in Abuja. By concentrating on such problems, the work will be able to contribute into the developing body of the research on foodborne pathogens within the third world, specifically Africa.

This knowledge is instrumental in coming up with effective food safety protocols to reduce the danger of anti-microbial resistance and protect the population of Nigeria. The study therefore provides a unique concept by combining the vendor practice with microbiological analysis contributing to a comprehensive view of safety of spices in Nigeria.

## 2 Materials and methods

### 2.1 Study design

The study utilized a cross-sectional and experimental design. The cross-sectional design was employed for sample collection and prevalence assessment, while the experimental laboratory component was used to evaluate the antimicrobial susceptibility. Samples of natural spices, such as cloves, cinnamon, turmeric, rosemary and basil, were collected from different sources in local markets.

### 2.2 Study area

**Figure 1:**
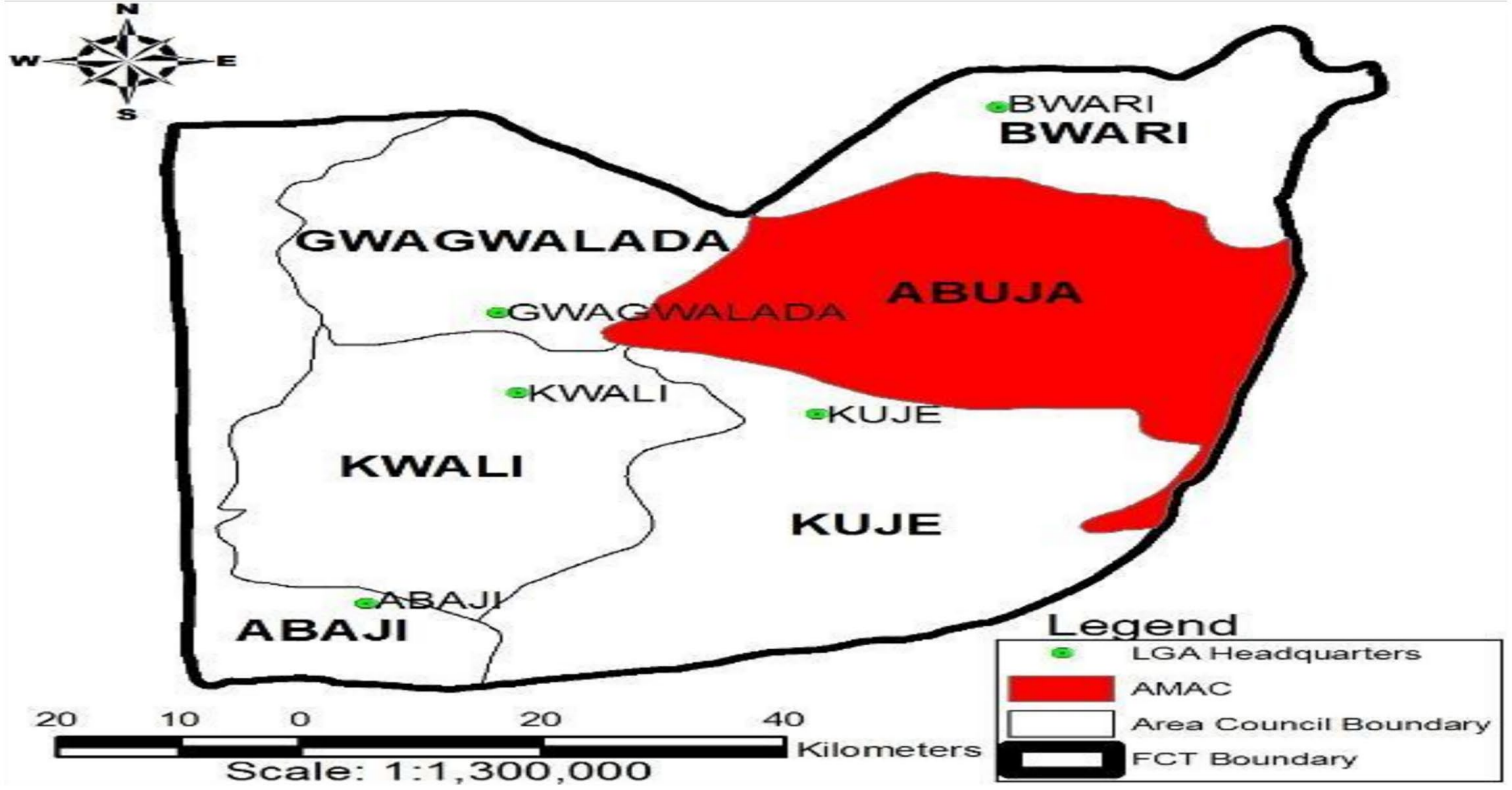
Map of Abuja municipal area council adapted from Mashi and Shuaibu (2018).

The Abuja Municipal Area Council (AMAC) is located on the eastern wing of the Federal Capital Territory (Figure 1). The bulk of federal institutions, ministries, and embassies are located within the confines of the Area Council. The Area Council is accessible through land, air, and telecommunication. The market holds several public and private bus terminals. The major means of transportation within the town are the motor cycle (Okada) and tricycle (Keke). It has a high influx of Hausa community and people from far and near areas, which comes with different food spices, such as cloves, cinnamon, turmeric, rosemary and basil. All major markets in the town are under the supervision of the local government, with designated shops for food spice sellers used for this study. Among the markets in AMAC, Nyanya Market was selected because, it serves as a major hub for the sale and distribution of spices in Abuja, Nigeria. According to the media report, the market attracts customers from different parts of Abuja and beyond (The Sun, 2025). It represents a critical point of potential microbial exposure due to high human traffic, storage conditions, and handling practices.

### 2.3 Study population

The study population included 800 natural (plant-based) spice sellers in major markets in Abuja (Nyanya), which include cloves, cinammon, turmeric, coriander seeds, rosemary, cumin, nutmeg and ginger powder sellers. However, a list of all the major markets in Nyanya town was obtained from the Abuja Municipal Area Council (AMAC) department in charge of market supervision.

### 2.4 Sample size and sampling method

#### 2.4.1 Sample size

A sample size of 267 natural spice sellers (cloves, cinammon, turmeric, coriander seeds, rosemary, cumin, nutmeg and ginger) in Abuja (Nyanya) town major markets was used for the purpose of this study. This sample size was determined by the Taro Yamane sample size formula for food stuffs in the area under study (Yamane, 1967), with a confidence interval of 95% and a standard error of 5%. The statistical formula n = $\frac{N}{1+N(e)2}$ was used.

Where *n* signifies the sample size, *N* signifies the population under study, e signifies the margin error and 1 is the constant.

The population of food stuff sellers associated with various markets in the area of study is 800, where 1 is a constant and the error margin is set at 0.05.

Therefore, $\frac{800}{1+800\;(0.05)2}$


$$\frac{800}{1+800\;(0.0025)}\frac{800}{1+2}\frac{800}{3}=266.7\;\text{appro}.=267$$

The sample size for this study was 267 plant-based food spice sellers.

#### 2.4.2 Sampling technique

A stratified random sampling technique was deployed in the first stage, where the target population was grouped into five strata, A B, C, D and E, on the basis of the lane locations of their shops. The study population included 800 natural (plant-based) spice sellers across the major market in Nyanya, Abuja Municipal Area Council (AMAC), Nigeria. Proportional sampling was performed, and the composition of each stratum was as follows: Lane A, 120 (15%); Lane B, 200 (25%); Lane C, 160 (20%); Lane D, 160 (20%); and Lane E, 160 (20%).

*Sample distribution*: The sample size of 267 sellers was then proportionally distributed across these lanes according to their population share, as shown below:

Lane A: (15% of 267) = 40 sellers.

Lane B: (25% of 267) = 67 sellers.

Lane C: (20% of 267) = 53 sellers.

Lane D: (20% of 267) = 53 sellers.

Lane E: (20% of 267) = 54 sellers.

Systematic sampling was used in the second stage to select the spice sellers. The proportion of each stratum was used to divide the sample size, and an interval of 3 was obtained for each stratum. During the administration of the questionnaire at the market and the meeting of the various spice sellers, simple random sampling was carried out to determine the entry point. Consequently, numbers 1–3 were written on paper, and each of the papers was folded and put in a small basket. The basket was shuffled, and one of the papers was drawn by a trained research assistant. Any number picked was used as the starting point, and 3 spice sellers’ intervals were maintained for the purpose of collecting the samples except where the third person refused to release the natural spices. The sample was subsequently collected from the next spice seller that was willing to give, solely for the study.

### 2.5 Method of data collection

#### 2.5.1 Questionnaire administration

The structured questionnaire used in this study was developed for this study. It has two sections, the section A has information on the sociodemographic characteristics of the natural spice sellers, while section B contained questions on the post-harvest management practices (sourcing, storage, handling, and quality). It was administered to 267 randomly selected natural spice sellers across the different lanes in the markets via a systematic sampling approach, which was conducted in two stages.

The researcher and trained research assistants approached the selected sellers, explained the purpose of the study, and obtained their consent before administering the questionnaire. The questionnaire was completed through face-to-face interviews. This method ensured that the selection of spice sellers was both random and representative of the overall population, reducing bias and increasing the reliability of the study results.

#### 2.5.2 Collection of natural spices

The following natural spices were obtained from the vendors: clove, cinammon, turmeric, coriander seeds, rosemary, cumin, nutmeg and ginger powder. The total number of samples obtained was 2,136. On average, 8 samples were obtained from each natural spice vendor according to the categories that comprised clove, cinammon, turmeric, coriander seeds, rosemary, cumin, nutmeg and ginger powder for the 267 spice vendors. Personal protective gear (PPE; i.e., lab coats and gloves) was worn before sample collection in order to maintain a sterile environment. Samples were collected aseptically via a sterile container to avoid further external contamination during collection. Each sample was labeled with the respective information, such as the name of the sampled spice, the location and date of collection and sealed immediately in a Ziploc bag and stored at the recommended temperature and transported in a storage facility by utilizing ice-pack and triple packaging system (Giostyle cooling box) to ensure samples’ quality and integrity.

### 2.6 Microbiological analysis

The inoculation of natural spices into sterile Petri dish plates and identification of bacteria were performed aseptically at the Laboratory of Infectious Disease and Molecular Epidemiology at the Department of Public Health, Federal University of Technology, Owerri, Imo State, Nigeria. First, the natural spices were separated on a Biosafety Cabinet II. One gram of sample was added to a sterile tube with 5 mL of Stuart transport medium, and the sample was allowed to wet completely. The tube was tightly closed to prevent leakage and contamination. The samples were vortexed and 1 mL was taken for tenfold serial dilution, and 90 μL of eluate was used to inoculate each plate per test condition (Ekeleme et al., 2024). The following culture media were inoculated into all samples: MacConkey agar with cefotaxime (Hardy), cetrimide agar (Hardy), mannitol salt agar (Hardy), Salmonella Shigella agar (Hardy), blood agar (Hardy), and chocolate agar (Hardy). In the negative control, the media plates in each batch were incubated uninoculated to ensure sterility (no growth). The positive controls were reference organisms obtained from the American Type Culture Collection (ATCC), Manassas, VA, USA, whose growth characteristics were known to confirm performance*: Escherichia coli* ATCC 25922 (MacConkey agar with cefotaxime), *Staphylococcus aureus* ATCC 25923 (mannitol salt agar and blood agar), *Pseudomonas aeruginosa* ATCC 27853 (cetrimide agar), and *Salmonella enterica serovar typhimurium* ATCC 14028 and *Shigella flexneri* ATCC 12022 (Salmonella–Shigella agar). In an air incubator, the plates were incubated at 37 °C for up to 48 h (Sukhum et al., 2022). Upon the establishment of growth on the culture media, the plates were counted using an electronic colony counter, the cfu/g was calculated by multiplying number of colonies counted by the dilution factor and divided by the volume plated and all the isolates were subcultured and purified and then stored at −80 °C in Tryptic Soy Broth with glycerol concentration of 20% (v/v). Isolated microorganisms were identified via a combination of morphological, biochemical, and microscopic examinations of colony morphology, cell shape, and size, which provided initial clues for identification (Ekeleme et al., 2020). The isolates were further subjected to genomic DNA extraction and purification was done using ZR DNA MiniPrep™ kit (50 Preps, Model D6005) from Zymo Research, California, USA according to the manufacturer’s instructions. DNA sequencing was performed by Sanger (dideoxy) Sequencing Technique to determine the nucleotide sequence of the specific bacteria isolated using automated PCR cycle-Sanger Sequencer™ 3730/3730XL DNA Analyzers from Applied Biosystems (Russell, 2002; Metzenberg, 2003). The amplification of the 16S rRNA gene was performed using universal bacterial primers: 27F (Forward Primer): 5′ AGAGTTTGATCMTGGCTCAG–3′ and 1492R (Reverse Primer): 5′ TACGGYTACCTTGTTACGACTT–3′. Meanwhile, a total of 20 percent of the total isolates were randomly chosen among the samples and was sent to an independent laboratory (Nigerian Institute of Medical Research, Yaba, Lagos, Nigeria) for reanalysis which confirmed the reproducibility of findings. The agreement of the findings in the two laboratories also validated the reproducibility and validity of the findings, and this in turn enhanced the validity of the study and served as an external validation measure.

### 2.7 Antimicrobial susceptibility testing

Antimicrobial susceptibility testing was performed for the identified bacteria via standardized methods, such as single disc diffusion, as described by the National Committee for Clinical and Laboratory Standard Institute [National Committee for Clinical and Laboratory Standard Institute (NCCLS), 2021]. Bacteria were grown on nutrient broth at 37 °C for 18–24 h. The suspension was visually adjusted with normal saline such that it was equal to 0.5 MacFarland turbidity standards. A sterile cotton-tipped applicator was introduced into a standardized inoculum and used to inoculate dried plates of Mueller–Hinton agar (Oxoid, England). Single sterile antibiotic discs were placed on each plate and incubated for 24 h aerobically at 37 °C. The antibiotics (Hardy Diagnostics) used were ciprofloxacin (10 μg, CPX), erythromycin (10 μg, E), ampicillin-sulbactam (30 μg, SAM), cefotoxine (10 μg, CTX), tetracycline (30 μg, TE), ampicillin (10 μg, AMP), Amoxicillin (AMX, 10 μg), Ceftriaxone (CRO, 30 μg), Gentamicin (GN, 10 μg), Vancomycin (VAN, 30 μg), and Chloramphenicol (C, 30 μg). The diameter of the zone of inhibition of each isolate to the disc was read with a calibrated ruler according to the standardized CLSI 2021 [Clinical and Laboratory Standards Institute, 2021] guidelines [National Committee for Clinical and Laboratory Standard Institute (NCCLS), 2021]. The zone of inhibition for each antimicrobial agent was documented. The susceptibility patterns were analysed to identify any prevalent antimicrobial resistance among the microorganisms isolated from the natural spices.

### 2.8 Method of data analysis

All the data obtained via the questionnaire were coded and entered into the Statistical Package for Social Sciences (SPSS version 21) for analysis. Descriptive statistics such as frequencies and percentages were used to summarize the data. Chi-square tests (*χ*^2^), were applied to determine associations between variables, including socio-demographic characteristics, post-harvest management practices, and the mean bacterial count. Normality was assessed using the Shapiro–Wilk test and homogeneity of variance using Levene’s test. Differences in mean bacterial counts among spice types (*n* = 267 per spice) were evaluated using one-way ANOVA. Pairwise comparisons were performed using Tukey’s HSD *post-hoc* test. Statistical significance was set at *p* < 0.05.

### 2.9 Results

This result presents the natural spices used for culinary purposes in Nigeria: vendor practices, bacterial contamination, and antimicrobial susceptibility patterns.

Table 1 shows the distribution of the demographic information of the 267 spice vendors interviewed in the Nyanya Market, Abuja. Male vendors stand at 24%, whereas female vendors stand at 76% of the respondents. The largest number of respondents were from the 30–39 years age group at 35%, the second lowest was from the 20–29 years age group at 22%, and the lowest age group was 60 years and above at 3%. Marital status shows that married sellers account for 67% of the respondents, suggesting that most vendors are family-oriented. The single vendors contributed 25% of the total, whereas the divorced and widowed sellers contributed only 5 and 3%, respectively. Most vendors had a secondary education, representing 44% of the respondents; 37% had received primary education, indicating that many vendors are literate. According to the survey, only 3% of them have a tertiary education, indicating that the vendors have limited access to education. Most respondents had 4–6 years of experience in the business of selling spices, which accounted for more than one-third of the entire returned sample. What has become very conspicuous is that only 20% have 1–3 years or 7–10 years of working experience. Only 10 percent of the respondents had been in business for less than 1 year, indicating that there were some new entrants into the business.

**Table 1:** Sociodemographic characteristics of the natural spices sellers (respondents).

Category	Frequency (*n*)	Percentage (%)
Gender		
Male	65	24
Female	202	76
Age group		
Under 20 years	13	5
20–29 years	59	22
30–39 years	93	35
40–49 years	67	25
50–59 years	27	10
60 years and above	8	3
Marital status		
Single	67	25
Married	179	67
Divorced	13	5
Widowed	8	3
Level of education		
No formal education	42	16
Primary education	99	37
Secondary education	117	44
Tertiary education	9	3
Years of experience in selling spices		
Less than 1 year	27	10
1–3 years	53	20
4–6 years	93	35
7–10 years	53	20
More than 10 years	40	15%

Table 2 shows that 62% of spices are sourced from other regions in Nigeria, with 45% of sellers obtaining them from middlemen. Most vendors (52%) sell daily, with 35% storing spices for 3–4 weeks. Common storage practices include open air (40%) and covered containers (32%). Handling is mostly done with bare hands (45%), and only 15% use gloves. While 60% of vendors practice hygiene, only 15% receive training on spice handling. Quality control was performed primarily through visual inspection (50%), but antimicrobial properties were not verified in 92% of the samples. Eighty-eight percent reported no customer complaints, with storage/handling affecting spice quality for 75%.

**Table 2:** The post-harvest management practices (sourcing, storage, handling, and quality).

Category	Frequency (*n*)	Percentage (%)
Origin of spices		
Locally sourced (within Abuja)	75	28
Sourced from other regions in Nigeria	166	62
Imported from other countries	26	10
How spices are sourced		
Directly from farmers	67	25
From middlemen/wholesalers	120	45
From other markets	75	28
Other	5	2
Frequency of spice sales		
Daily	139	52
Weekly	93	35
Biweekly	27	10
Monthly	8	3
Storage duration of spices		
Less than a week	53	20
1–2 weeks	80	30
3–4 weeks	93	35
More than a month	41	15
Type of storage practices		
Open air (stalls/tables)	107	40
Covered containers	85	32
Refrigeration	8	3
Shelves in closed shops	67	25
Handling practices		
Use of bare hands	120	45
Use of gloves	40	15
Use of utensils (spoons, scoops)	80	30
Prepackaged	27	10
Hygiene practices		
Yes	160	60
No	107	40
Received training on food safety/spice handling		
Yes	40	15
No	227	85
Frequency of quality checks		
Always	80	30
Sometimes	133	50
Rarely	40	15
Never	14	5
Quality control measures employed		
Visual inspection for contamination	133	50
Smell test	80	30
Sorting out damaged/spoiled spices	27	10
Moisture content testing	13	5
Other	14	5
Verifying antimicrobial properties		
Yes	21	8
No	246	92
Customer complaints		
Yes	32	12
No	235	88
Handling complaints		
Direct refund/replacement	11	40
Apologize and educate	8	30
Dismiss complaints	4	15
Investigate and adjust practices	4	15
Impact of storage/handling on spice quality		
Yes	200	75
No	67	25
Suggestions for improving spice quality/safety		
Provide training on spice handling	93	35
Use better storage facilities	120	45
Conduct regular quality checks	40	15
Improve packaging	14	5

Table 3 shows that within each column, values with different superscript letters differ significantly at *p* < 0.05 based on one-way ANOVA followed by Tukey’s HSD *post-hoc* test. Cinnamon recorded the lowest total viable count (0.1 × 10^4^ CFU/g), the lowest coliform count (1.0 × 10^2^ CFU/g), and a low total bacterial count (1.5 × 10^3^ CFU/g), suggesting that cinnamon has strong antimicrobial properties.

**Table 3:** Mean bacteria count associated with natural spices.

Spice (*n* = 267 each)	Total viable count (cfu/g) × 10^4^	Coliform count (cfu/g) × 10^2^	Total bacteria count (cfu/g) × 10^3^
Cloves	1.2 ± 0.3^b^	5.0 ± 0.1^c^	3.4 ± 0.1^c^
Cinnamon	0.1 ± 0.5^a^	1.0 ± 0.1^a^	1.5 ± 0.2^a^
Turmeric	3.4 ± 0.2^e^	8.0 ± 0.5^e^	4.0 ± 0.1^d^
Coriander seeds	0.2 ± 0.1^a^	6.0 ± 0.3^d^	2.5 ± 0.2^b^
Rosemary	0.2 ± 0.1^a^	2.0 ± 0.2^b^	1.0 ± 0.1^a^
Cumin	1.6 ± 0.3^c^	7.5 ± 0.1^e^	2.0 ± 0.3^b^
Nutmeg	2.2 ± 0.2^d^	5.5 ± 0.2^c^	3.0 ± 0.1^c^
Ginger	3.0 ± 0.2^e^	9.0 ± 0.3^f^	4.5 ± 0.4^d^

Data are presented as mean ± SE; *n* = 267 independent samples per spice. Differences among spices were assessed using one-way ANOVA with Tukey’s HSD *post-hoc* test; significance was set at *p* < 0.05. Within each column, values with different superscript letters differ significantly at *p* < 0.05.

Clove had a moderate total viable count (1.2 × 10^4^ CFU/g) and coliform count (5.0 × 10^2^ CFU/g), with a total bacterial count of 3.4 × 10^3^ CFU/g, indicating a fair bacterial presence but not at alarming levels.

Turmeric had one of the highest total viable counts (3.4 × 10^4^ CFU/g), a coliform count of 8.0 × 10^2^ CFU/g, and a total bacterial count of 4.0 × 10^3^ CFU/g, suggesting it was one of the most contaminated spices.

The correlation between socio-demographic characteristics, post-harvest management practices, and the mean bacterial count using multivariate linear regression analysis was statistically significant (*F*(8, 2,127) = 10.27, *p* < 0.001), explaining 64% of the variance in mean bacteria count (*R*^2^ = 0.64; adjusted *R*^2^ = 0.58). Storage type (*β* = −0.298, *p* = 0.042), storage duration (*β* = 0.431, *p* = 0.008), handling practice (*β* = −0.210, *p* = 0.043), and quality checks (*β* = −0.512, *p* = 0.009) were significant predictors. No evidence of multicollinearity was detected, as VIF values ranged from 1.18 to 1.83, below the recommended threshold of 5 (Table 4).

**Table 4:** Multivariate regression analysis of factors affecting mean bacteria count.

Variable	Coefficient (*β*)	Std. error	*t*-value	*p*-value	VIF
Constant	4.215	1.103	3.82	0.005**	0.0
Gender (male = 0, female = 1)	0.023	0.112	0.21	0.835	1.18
Age	−0.027	0.015	−1.80	0.109	1.42
Education level	0.420	0.121	3.48	0.084	1.67
Years of experience	−0.045	0.027	−1.67	0.133	1.59
Storage type	−0.298	0.142	−2.10	0.042	1.74
Storage duration	0.431	0.120	3.59	0.008**	1.83
Handling practice	−0.210	0.109	−1.92	0.043	1.61
Quality checks	−0.512	0.144	−3.56	0.009**	1.76

**Significant (*p* < 0.05), *R*^2^ = 0.64; adjusted *R*^2^ = 0.58; *F* = 10.27, multicollinearity showed acceptable levels (mean VIF = 1.60; max VIF = 1.83).

Table 5 shows that turmeric (18.4%) and cumin (17.1%) had the highest contamination levels, reflecting higher vulnerability to microbial growth. Coriander (9.7%) and nutmeg (8.6%) had the lowest rate of contamination with fewer bacterial population. Overall, 7.1% of all spice samples purchased were positive for bacterial isolates, while 92.9% were microorganism-free of interest.

**Table 5:** Prevalence of microbial contamination in different natural spice samples collected from Nyanya Market, Abuja.

Spice	Total samples collected	Positive for microorganisms (%)	Negative for microorganisms (%)
Cloves	267	17 (11.2)	250 (88.8)
Cinnamon	267	16 (10.5)	251 (89.5)
Turmeric	267	28 (18.4)	239 (81.6)
Coriander seeds	267	15 (9.9)	252 (90.1)
Rosemary	267	21 (13.8)	246 (86.2)
Cumin	267	26 (17.1)	241 (82.9)
Nutmeg	267	13 (8.6)	254 (91.4)
Ginger	267	16 (10.5)	251 (89.5)
Total	2,136	152 (7.1)	1984 (92.9)

The distribution of bacterial isolates across various natural spices is presented in Table 6. Turmeric had the greatest number of bacterial isolates, accounting for 28 (18.4%) of the total, indicating significant bacterial diversity. Cumin followed with 26 (17.1%) isolates, also indicating high bacterial presence and Nutmeg had the lowest bacterial presence, with 13 (8.6%) isolates.

**Table 6:** Distribution of bacterial isolates among the different types of natural spices.

Spice bacteria	Cloves (%)	Cinnamon (%)	Turmeric (%)	Coriander seeds (%)	Rosemary (%)	Cumin (%)	Nutmeg (%)	Ginger (%)
*Escherichia coli* (*n* = 17)	1 (5.9)	3 (17.6)	4 (23.5)	1 (5.9)	4 (23.5)	2 (11.8)	1 (5.9)	1 (5.9)
*Staphylococcus aureus* (*n* = 19)	2 (10.5)	2 (10.5)	3 (15.8)	2 (10.5)	3 (15.8)	3 (15.8)	2 (10.5)	2 (10.5)
*Klebsiella pneumoniae* (*n* = 17)	1 (5.9)	3 (17.6)	3 (17.6)	3 (17.6)	2 (11.8)	2 (11.8)	1 (5.9)	2 (11.8)
*Bacillus subtilis* (*n* = 16)	4 (25)	1 (6.2)	2 (12.5)	1 (6.2)	1 (6.2)	4 (25.0)	1 (6.2)	2 (12.5)
*Pseudomonas aeruginosa* (*n* = 16)	1 (6.2)	1 (6.2)	4 (25)	1 (6.2)	2 (12.5)	4 (25)	2 (12.5)	1 (6.2)
*Salmonella* spp. (*n* = 16)	1 (6.2)	1 (6.2)	3 (18.8)	1 (6.2)	2 (12.5)	4 (25)	1 (6.2)	3 (18.8)
*Enterococcus faecalis* (*n* = 10)	2 (20.0)	1 (10.0)	3 (30.0)	0 (0.0)	1 (10.0)	1 (10.0)	1 (10.0)	1 (10.0)
*Bacillus cereus* (*n* = 18)	3 (16.7)	2 (11.1)	4 (22.2)	1 (5.6)	4 (22.2)	2 (11.1)	1 (5.6)	1 (5.6)
*Staphylococcus epidermidis* (*n* = 14)	1 (7.1)	1 (7.1)	1 (7.1)	3 (21.4)	1 (7.1)	3 (21.4)	2 (14.3)	2 (14.3)
*Enterobacter* spp. (*n* = 9)	1 (11.1)	1 (11.1)	1 (11.1)	2 (22.2)	1 (11.1)	1 (11.1)	1 (11.1)	1 (11.1)
Total (*n* = 152)	17 (11.2)	16 (10.5)	28 (18.4)	15 (9.7)	21 (13.8)	26 (17.1)	13 (8.6)	16 (10.5)

*Escherichia coli* (*n* = 17) was most frequently found in Turmeric and Rosemary (23.5% each). *Staphylococcus aureus* (*n* = 19) was equally distributed across Turmeric, Rosemary, and Cumin (15.8% each). *Klebsiella pneumoniae* (*n* = 17) relatively even spread, particularly in Cinnamon, Turmeric, and Coriander seeds (17.6% each). *Bacillus cereus* (*n* = 18) was most common in Turmeric and Rosemary (22.2% each). *Staphylococcus epidermidis* (*n* = 14) and *Enterobacter* spp. (*n* = 9) were distributed more evenly across spices, with the highest presence of *S. epidermidis* in Coriander seeds and Cumin (21.4% each.)

Table 7 shows the antimicrobial susceptibility profile of the bacteria isolates associated with natural spices. The zone of inhibition for ciprofloxacin had the highest against *E. coli,* with a zone of inhibition of 25 mm, whereas the zone of inhibition for gentamicin was 22 mm. *Escherichia coli* was the most sensitive to antibiotics but relatively more resistant to ampicillin, with a zone of inhibition of 9 mm, and tetracycline, with a zone of inhibition of 10 mm. *Staphylococcus aureus* was the most sensitive to ciprofloxacin (20 mm) and gentamicin (18 mm) and the least sensitive to ampicillin (7 mm) and tetracycline (9 mm). *Escherichia coli* is susceptible to ciprofloxacin (CPX) and gentamicin (GN) but resistant to ampicillin (AMP). *Bacillus subtilis* and *B. cereus* are susceptible to most antibiotics. *Staphylococcus aureus* is resistant to ampicillin, vancomycin (VAN), and erythromycin (E). *Klebsiella pneumoniae* is susceptible to ciprofloxacin, gentamicin, and ceftriaxone (CRO) but resistant to tetracycline (TE). This pattern highlights the varying effectiveness of antibiotics against different bacteria associated with natural spices.

**Table 7:** Antimicrobial susceptibility profile of the bacteria isolates associated with natural spices.

Antibiotics/zones of inhibition (mm)										
Microbial isolates	CPX (10 μg)	AMX (10 μg)	CRO (30 μg)	TE (30 μg)	GN (10 μg)	AMP (10 μg)	VAN (30 μg)	C (30 μg).	E (10 μg)	CTX (10 μg)
*Escherichia coli*	25	10	17	10	22	9	15	12	14	19
*Staphylococcus aureus*	20	10	15	9	18	7	12	9	11	17
*Klebsiella pneumoniae*	18	9	16	9	20	10	13	10	14	20
*Bacillus subtilis*	21	10	18	22	21	18	20	22	19	21
*Pseudomonas aeruginosa*	20	13	19	10	12	11	17	13	15	20
*Salmonella* spp.	21	19	20	12	18	9	18	12	16	21
*Enterococcus faecalis*	20	15	18	11	14	12	18	11	13	19
*Bacillus cereus*	21	13	19	20	21	17	19	23	19	22
*Staphylococcus epidermidis*	21	15	20	17	19	8	13	10	11	18
*Enterobacter* spp.	19	16	20	10	19	10	14	13	15	15

CPX, ciprofloxacin; AMX, amoxicillin; CRO, ceftriaxone; TE, tetracycline, GN, Gentamicin; AMP, ampicillin; VAN, vancomycin; C, chloramphenicol, E, erythromycin; CTX, cefotoxine; Green = susceptible (S); Yellow = Intermediate (I); Red = Resistant (R).

**Figure 2:**
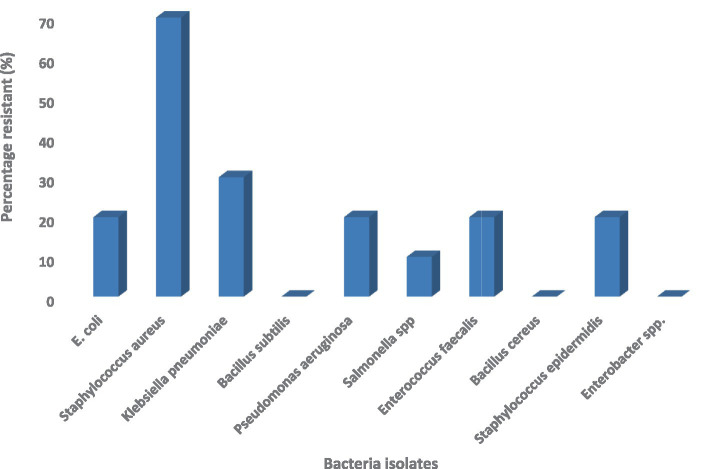
Antimicrobial resistance pattern of bacterial isolates from natural spices.

Figure 2 shows the percentage of resistant isolates (%) R, as the value calculated using the Kirby-Bauer disc diffusion method and interpreted based on CLSI M100 (2024) breakpoints. The greatest resistance was observed in *S. aureus* (70%), and the lowest *in B. subtilis, B. cereus* and *Enterobacter* spp. (0%). Percent values are the percentage of resistant isolates in comparison to the number of isolates used as a specimen of a particular species.

## 3 Discussion

The study highlights significant microbial contamination in natural spices sold in Nyanya Market, Abuja, emphasizing the implications for food safety and public health. The presence of bacteria such as *E. coli*, *S. aureus*, *K. pneumoniae*, *B. cereus*, and *Pseudomonas aeruginosa* raises concerns about spice handling, storage, and potential health risks. Contamination levels varied among spices, with turmeric and ginger exhibiting the highest microbial loads, while cinnamon and rosemary showed the lowest levels, likely due to their antimicrobial properties (Ibrahim et al., 2021). The contamination of the spices undermines their antibacterial and antifungal activities against food spoilage bacteria like *B. subtilis*, *Pseudomonas fluorescens*, *S. aureus* and *Vibrio parahaemolyticus* (Liu et al., 2017). The antimicrobial resistance patterns observed in this study align with the growing burden of AMR reported across Nigeria. Regional surveillance studies have consistently documented the overall pooled prevalence of ESBL-PE in Nigeria was 34.6% (95% CI 26.8 to 42.3%) and increased at a rate of 0.22% per year (*p* for trend = 0.837) (Musa et al., 2020).

The hygiene and storage practices of spice vendors significantly influenced microbial contamination. Vendors with limited education (16%) may lack awareness of proper food safety measures, increasing the risk of contamination through poor handling, inadequate storage, and exposure to environmental contaminants (Ibrahim et al., 2020). A majority (45%) handled spices with bare hands, and only 15% used gloves, heightening the risk of cross-contamination (Adefolalu and Johnson, 2021). Storage duration also played a role—spices stored for over 3 weeks had higher microbial loads, emphasizing the need for improved preservation techniques and vendor training (Adeyemi and Okoli, 2019; Akinyemi et al., 2020).

The contamination of natural spices by microorganisms significantly relies on storage duration, vendor training, and quality testing (Oyeleke and Manga, 2020), with longer storage duration increasing bacterial load (*p* = 0.008) while regular monitoring reduces it (*p* = 0.009). Antimicrobial-resistant (AMR) bacterial reservoirs are caused by improper handling and storage resulting in serious food safety risks (Ibrahim et al., 2021). Antimicrobial-resistant *E. coli* and *S. aureus* isolates were isolated, leading to AMR risks through foodborne transmission (Adegoke et al., 2022). Enhanced hygiene practices, regulated storage, and surveillance of AMR are essential in reducing public health threats from infected species (Cicero et al., 2022; Kyrylenko et al., 2023).

The bacteria isolated from the spices such as *S. aureus* exhibited high resistance to ampicillin (AMP), vancomycin (VAN), and erythromycin (E), indicating the presence of multidrug-resistant strains (Adegoke et al., 2022; Allahghadri et al., 2010). Similarly, *E. coli* and *K. pneumoniae* showed resistance to tetracycline (TE) and amoxicillin (AMX), reflecting regional antibiotic misuse and selection pressure (Bauer et al., 2014; Angel et al., 2014). These findings align with global AMR trends, where overuse of antibiotics in human health and agriculture has contributed to the rise of resistant pathogens in food products (Gera et al., 2017).

The contamination of spices with AMR bacteria poses direct risks to public health (Salam et al., 2023). Consumers may unknowingly ingest pathogenic and antibiotic-resistant bacteria, leading to foodborne infections that are harder to treat. Additionally, spices can act as vehicles for resistant genes, facilitating the spread of AMR through the food chain (Abd El Mageed et al., 2012; Bozin et al., 2007). The study underscores the need for strict regulatory oversight, improved hygiene education for spice vendors, and surveillance of microbial contamination in food products to reduce the burden of foodborne diseases and antimicrobial resistance.

The reported high levels of microbial loads and patterns of antimicrobial resistance in the chosen samples of spices highlight regulatory applicability of the NAFDAC Act CAP N1 (2004) specifically in Sections 5(a)–(e) that stipulate the quality of food, its inspection, and post-market surveillance. The results also correspond to the National Action Plan on Antimicrobial Resistance (2020–2025) in Nigeria, which underlines the necessity to integrate the spice products into the national AMR surveillance frameworks within a One Health framework. It is suggested that enhanced microbial standards, vendor hygiene certification, risk-based surveillance, and improved packaging regulations can be used to deter such contamination and the spread of antimicrobial resistance in the food chain.

### 3.1 Conclusion

In summary, these findings demonstrate the wide range of bacterial contamination across various natural spices, with some spices, particularly turmeric, being more susceptible to contamination than others. The presence of potentially pathogenic bacteria such as *E. coli*, *S. aureus*, and *B. cereus* raises significant food safety concerns. These findings highlight the need for improved hygiene practices, better storage conditions, and thorough cooking to minimize the risk of foodborne illnesses associated with contaminated spices.

### 3.2 Recommendation

These findings highlight the need for better handling and storage practices. With 45% of vendors using bare hands and only 15% receiving training in spice handling, more comprehensive hygiene training programs should be introduced to reduce contamination and improve food safety. Additionally, encouraging the use of gloves and better storage containers will limit bacterial growth and preserve spice quality. The high resistance rates of bacteria such as *S. aureus* and *K. pneumoniae* to commonly used antibiotics call for targeted antimicrobial strategies. Collaborative efforts between market authorities and healthcare agencies are needed to address this public health concern.

### 3.3 Contribution to knowledge

The study revealed that the prevalent use of open-air storage and the use of bare hands in the handling of spices poses risks of contamination. The detection of *E. coli*, *S. aureus*, and *K. pneumoniae* is of vital concern with respect to food safety within the spice trade. This finding provides practical information related to the resistance patterns of bacteria linked with natural spice antimicrobials, with *S. aureus* and *K. pneumoniae* showing significant resistance to widely used antibiotics, indicating a targeted approach toward combating resistant strains via specific antimicrobial agents.

## Data Availability

The datasets presented in this study can be found in online repositories. The names of the repository/repositories and accession number(s) can be found at: https://papers.ssrn.com/sol3/papers.cfm?abstract_id=5070377.
